# Closed head injury combined with orbital blowout fracture and displacement of the eyeball into the maxillary sinus in a 14-year-old boy: a case report

**DOI:** 10.1186/s12886-024-03421-w

**Published:** 2024-04-03

**Authors:** Yue Fu, Ying He, Huixuan Xie, Kongliang Sun, Hanjun Dai

**Affiliations:** 1 Department of Ophthalmology, Zhongnan Hospital of Wuhan University, Wuhan, China; 2 Department of Colorectal and Anal Surgery, Zhongnan Hospital of Wuhan University, Wuhan, China

**Keywords:** Orbital blowout fracture, Displacement of the eyeball, Teenager, Maxillary sinus, Case report

## Abstract

**Background:**

Trauma-induced orbital blowout fracture (OBF) with eyeball displacement into the maxillary sinus is rare.

**Case presentation:**

We present the case of a 14-year-old with a closed head injury, OBF, and displacement of the eyeball into the maxillary sinus following a car accident. A prompt transconjunctival access surgery was performed for eyeball repositioning and orbital reconstruction in a single session, mitigating anaesthesia-related risks associated with multiple surgeries. At the 12-month follow-up, his visual acuity was 20/200. Despite limited eye movement and optic nerve atrophy, overall satisfaction with the ocular appearance was achieved.

**Conclusions:**

This report offers novel insights into the mechanisms of OBF occurrence and the development of postoperative complications.

## Background

The orbit is a four-sided pyramidal bony cavity, oriented with its opening forward, comprising seven bones forming the upper, lower, inner, and outer walls. The average adult orbit volume in the Chinese population is approximately 28 mL; it is 13 mL for newborns. The orbit’s size grows linearly from ages 0 to 6, gradually increasing, reaching about 95% or more of the adult volume by age 12 [1–3]. The orbit accommodates vital tissues and organs, including the eyeball, extraocular muscles, lacrimal gland, blood vessels, optic nerve, adipose tissue, and fascia. The extraocular muscles regulate eyeball movement, while the optic nerve transmits visual information to the brain. Surrounding blood vessels supply the eyeball and related tissues, ensuring normal metabolism and function. Damage to any of these structures can potentially affect appearance or visual function.

Orbital blowout fracture (OBF) was initially named by Smith and Regan in 1957 [4]. Two primary hypotheses have been proposed to explain its mechanism. Smith and Regan’s theory, mainly involving “fluid malleability,” suggests that impact on the eyeball causes a rapid increase in intraorbital pressure, leading to fractures at weakened areas of the orbit. Alternatively, Fujino’s “entrapment mechanism” proposes that direct external force on the orbital rim, or even the skull, transmits force along the bone, causing instantaneous bending and deformation of the orbital wall, resulting in fractures at vulnerable points [5]. When the impact force ceases, the bone returns to its original position, while soft tissues—and even the eyeball—may be trapped within the fracture due to inertia.

Due to the greater presence of cancellous bone and cartilage in paediatric bones, the facial bones of children exhibit heightened elasticity and flexibility. Therefore, when a child’s orbit is subjected to external force, it is more susceptible to fissure-type greenstick fractures or trapdoor fractures, as opposed to extensive defects. As children grow, the patterns of orbital fractures change. Hink et al. suggests that children under 8 years are more prone to orbital roof fractures, whereas children aged 8 years or older predominantly experience orbital floor fractures [6]. Before age 8, the frontal sinus in children is incompletely air-filled, rendering the orbital roof susceptible to fractures from minor impacts. As the maxillary sinus develops and becomes fully aerated, the protection of the orbital floor diminishes, resulting in an increased incidence of orbital floor fractures. The extrusion or entrapment of orbital contents through the fracture gap can manifest as diplopia, restricted eye movement, and even eyeball displacement.

Among the various adverse outcomes of OBF, eyeball displacement and subsequent entrapment in the maxillary sinus are exceptionally rare [7]. A systematic literature search of English-language publications on PubMed revealed only 28 reported cases, with most complications being decreased vision, blurred vision, diplopia, and enophthalmos [8].

This article reports an extremely rare case involving a 14-year-old adolescent in a car accident with a closed head injury, OBF, and eyeball displacement embedded in the maxillary sinus. After actively stabilising the patient’s overall condition, surgical intervention was performed through transconjunctival access to reposition the eyeball and simultaneously reconstruct the orbit.

## Case presentation

A 14-year-old male was involved in a car accident, hitting the right side of his head on the ground. He immediately experienced impaired consciousness and projectile vomiting. He was promptly transported to the local hospital’s emergency department. Upon assessment, he presented with a Glasgow Coma Scale (GCS) score of 3. Computed tomography (CT) scans revealed a range of injuries, including comminuted fracture of the right frontal bone, contusion and laceration of the right frontal lobe, subarachnoid haemorrhage, potential right frontotemporal epidural haematoma, pneumocephalus, multiple fractures of the right orbit and bilateral sinus walls, fractures of bilateral maxillary bones, sphenoid bone, and nasal bone, along with damage and displacement of the right eyeball. Following assessment at the local hospital, an immediate craniotomy for intracranial haematoma evacuation and decompression under general anaesthesia was performed. The patient was transferred to the intensive care unit (ICU) post-surgery for monitoring and further treatment.

**Fig. 1:**
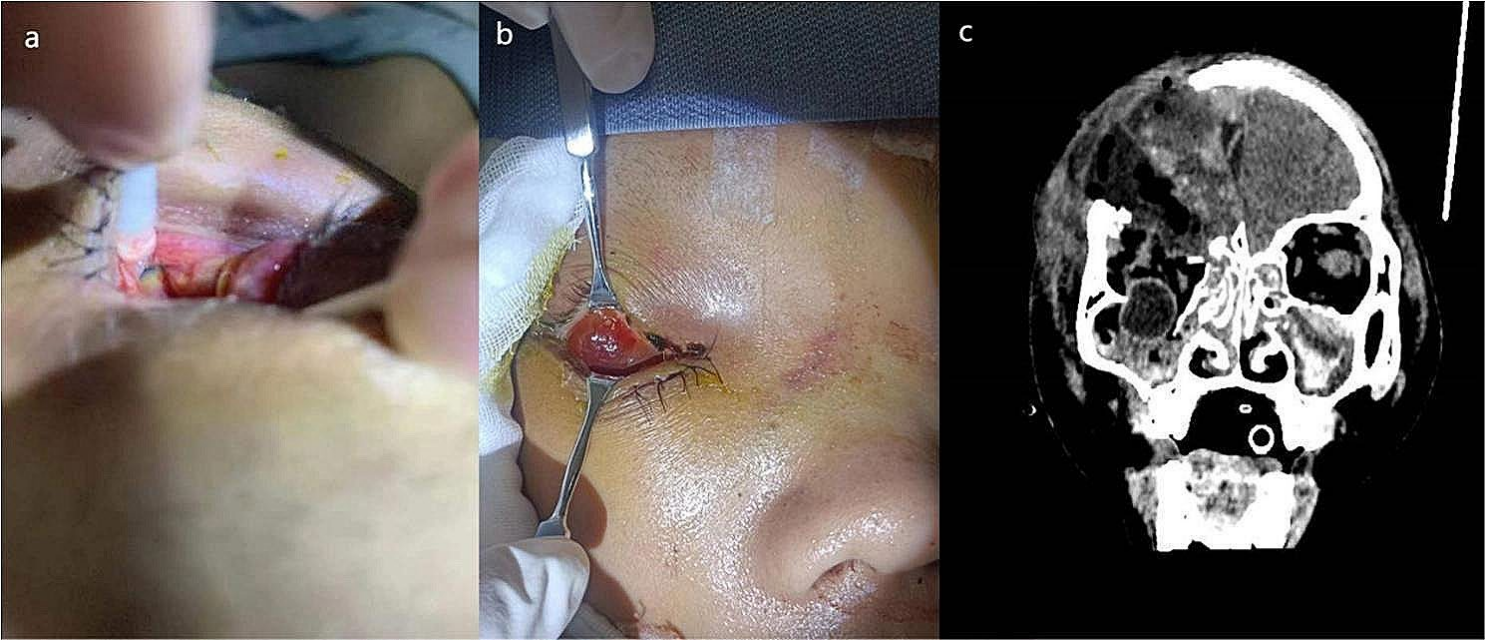
Right orbital cavity without eyeball on physical examination. (**a**). The dislocated eyeball could be observed from the patient’s head downwards. (**b**). Only conjunctival oedema was observed under direct vision. (**c**). Computed tomography showed extensive fractures of the orbital floor and eyeball displacement into the maxillary sinus

Due to limited resources at the local hospital, the patient was transferred to the ICU of the authors’ hospital for ongoing care 4 days post-surgery. Our initial assessment revealed that a portion of the right parietal bone was absent, and there was swelling of the skin on the right side of the face. The previously lacerated lower eyelid at the inner canthus had been sutured. On direct gaze, the right eyeball was missing from the lower orbit, and conjunctival oedema was observed. Retracting the eyeball and observing from the patient’s head downwards made the eyeball displacement apparent (Fig. 1a and b).

An orbit CT scan revealed extensive fractures of the orbital floor, with entrapment and deformation of the eyeball. The eyeball had partially shifted, entering the ipsilateral maxillary sinus (Fig. 1c). Following an initial assessment of the patient’s condition, it was deemed necessary to reposition the eyeball, requiring prompt surgical intervention. However, if the integrity of the eyeball had already been compromised, removing the right eye was also a potential consideration. Nevertheless, the patient was experiencing sustained high fever, with a peak temperature of 41℃, and blood culture indicated bacteraemia. The infection was attributed to multidrug-resistant *Klebsiella pneumoniae*. The patients had a GCS score of 3, a Richmond Agitation-Sedation Scale score of -4, and unstable vital signs. High doses of adrenaline were required to maintain vital signs, making it temporarily impractical for him to undergo another procedure involving general anaesthesia.

After comprehensive treatment, on the 8th day of admission, which marked the 12th day post-trauma, notable improvements were observed in the patient’s overall condition. Therefore, the ophthalmology team proceeded with a surgical intervention under general anaesthesia. Employing transconjunctival access, we gently separated the tissues from the inferior border of the orbital bone. Extensive comminuted fractures were evident from the orbit’s inferior border to the midsection of the orbital floor. The eyeball and fractured fragments had displaced into the maxillary sinus. We systematically removed the surrounding bone fragments, relieved pressure on the eyeball, and confirmed the integrity of the globe. The extraocular muscles were sequentially separated in the order of superior rectus, superior oblique, medial rectus, and lateral rectus. Upon completion of each extraocular muscle separation, traction sutures were anchored at the muscle insertion points of the rectus muscles to facilitate the separation of other extraocular muscles and ultimately guide the eyeball back into position. During the procedure, extraocular muscles were individually separated, revealing a tear in the right inferior rectus muscle, while the remaining extraocular muscles displayed a dark red colour with a compromised blood supply. Given the extent of the long-lasting consequences to the patient’s vital signs and extraocular muscles, eye movement impairment was anticipated.

Consequently, instead of seeking the torn end of the inferior rectus muscle, we used sutures anchored at the insertion points of the four rectus muscles to gently guide the eyeball back into position. Examination of the patient’s right eye revealed an intact structure, a dilated pupil, and a sluggish light reflex. The diameter of the pupil was 7 mm. An assessment of the bone defects revealed an absence of the orbital floor measuring 3 × 2.5 cm, a 2 cm deficiency in the orbital rim, and a 2 × 1.3 cm gap in the anterior upper part of the maxilla. To address these deficiencies, we implanted a MEDPOR TITAN (ORBITAL FLOOR AND WALL MTB RIGHT) (Stryker, Kalamazoo, MI, USA) prosthesis, approximately 3.1 × 2.6 cm in size (1.0-mm-thick), into the inferior orbital rim. The graft effectively covered the defect caused by the orbital floor fracture. As for the defect in the superior orbital wall, the brain surgeon convinced that the patient needed a second-stage skull base repair surgery in rid of leakage of cerebrospinal fluid after 6 months to 1 year. Skull base repair is more efficient than orbital roof repair to achieve better stability, so there is no need to repair the supraorbital wall during this operation.

Subsequent examination revealed no muscle or soft tissue entrapment, and the passive traction of the inferior, medial, and lateral rectus muscles was deemed satisfactory. Following the surgical intervention, the patient’s affected pupil retracted to a diameter of 2.5 mm, displaying a sluggish light reflex. The procedure lasted approximately 3 h, and eyeball repositioning and orbital reconstruction were successfully accomplished in a single session.

**Fig. 2:**
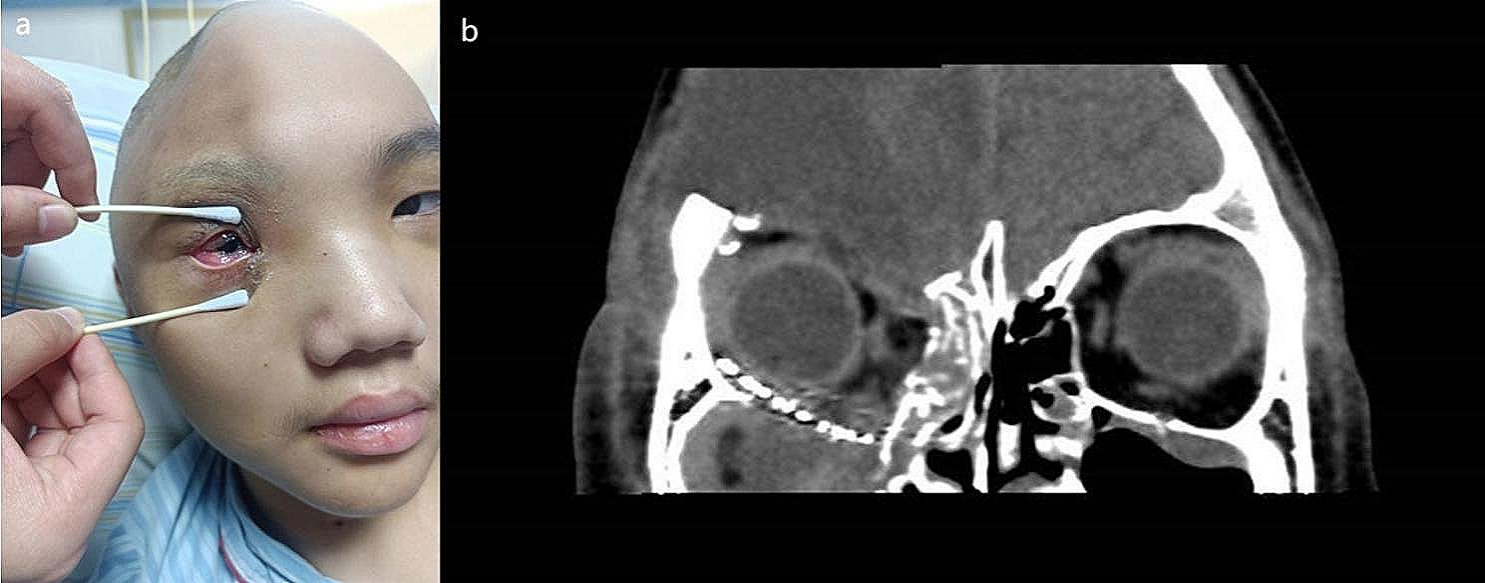
Postoperative examination. (**a**). Bedside examination revealed right eye conjunctival oedema, a pupil diameter of 2.5 mm, sluggish light reflex, and limited eye movement. (**b**). Computed tomography showed the titanium mesh in a satisfactory position with no entrapment of orbital tissues

**Fig. 3:**
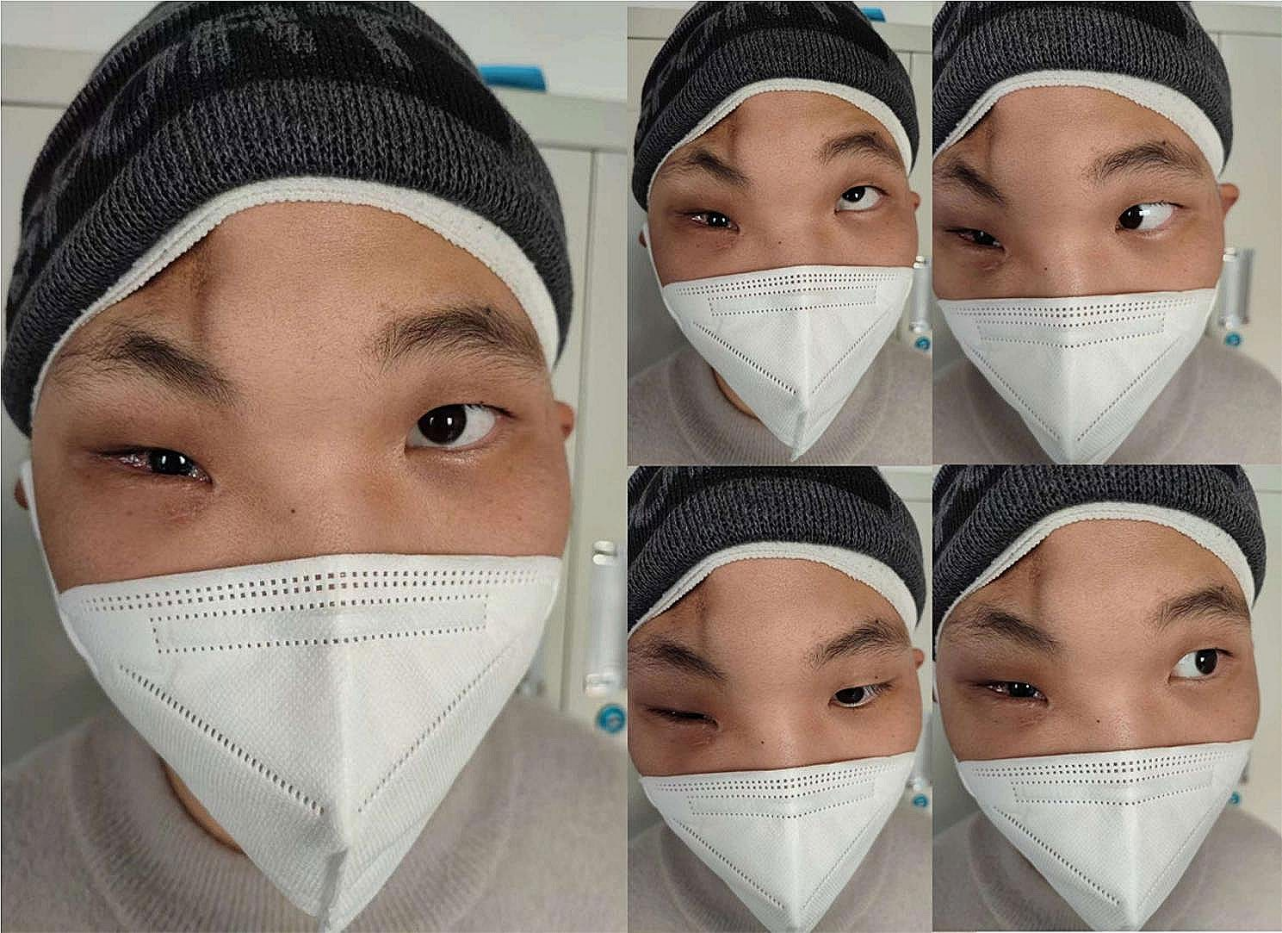
Restricted eye movement was observed 12 months postoperatively

The patient’s hospitalisation spanned 28 days, with discharge occurring 20 days post-surgery. Throughout this period, no steroid treatment was administered due to the patient’s young age. During a bedside eye examination, the right eye exhibited conjunctival oedema, a pupil diameter of 2.5 mm, sluggish light reflex, limited eye movement, and visual acuity of 40 cm (Fig. 2a). A follow-up CT scan before discharge showed the titanium mesh in a satisfactory position, with no entrapment of orbital tissues (Fig. 2b). At the 12-month postoperative assessment, a CT scan confirmed the well-positioned orbital reconstruction material without any entrapment of extraocular muscles or fat tissues. However, the visual acuity was 20/200, accompanied by restricted eye movement (Fig. 3). Slit lamp examination identified a traumatic cataract in the right eye, and macular optical coherence tomography (OCT) indicated retinal atrophy in the macular area of the right eye, along with alterations in the axial length of the eye. Optic nerve OCT revealed atrophy of the optic nerve in the right eye. The patient was generally satisfied with the treatment results.

## Discussion and conclusions

To the best of our knowledge, this represents the initial instance of successful treatment for a teenager presenting with a complex constellation of conditions including orbital blowout fracture, displacement of the eyeball with entrapment in the maxillary sinus, alongside a concomitant closed head injury stemming from trauma. Furthermore, this appears to be the most youthful patient on record with such a presentation. In this case, the patient’s condition was unique, marked by impaired consciousness, unstable vital signs, prolonged high fever, and a recent history of general anaesthesia for cranial surgery upon the initial assessment. This made an immediate application of a second general anaesthesia for addressing ophthalmic issues unfeasible. Consequently, his overall condition and vital signs represent the most severe among all documented cases in the literature.

For this patient, the impact initially occurred on the right frontal region, aligning with Fujino’s hypothesis [5] that the entrapment mechanism was pivotal in the fracture’s occurrence. The severe head injury experienced by the patient involved force transmitted through the bone structure, causing an instantaneous bending and deformation of the orbital wall, resulting in fractures at vulnerable points. Furthermore, the mechanism behind OBF in this patient involved a sharp increase in intracranial pressure, which played a significant role following the head impact. Fluids such as cerebrospinal fluid and blood circulate within the intracranial space. When the head undergoes a violent impact, the inertia of these fluids creates a pressure gradient. The rapidly elevated intracranial pressure is then transmitted through brain tissue to the base of the skull. This pressure gradient may lead to more prolonged and intense pressure injuries, bone destruction, and, consequently, multiple comminuted fractures in the orbital, nasal, ethmoid, and maxillary bones. Contusions in the frontal lobe and subarachnoid haemorrhage were also consequences of these pressure variations.

In cases where the eyeball is displaced and embedded in the maxillary sinus, the choice of surgical approach becomes pivotal in treatment decisions. Scholars frequently avoid transconjunctival access methods due to the potential risk of inducing secondary eye damage and the heightened surgical complexity associated with such approaches. Instead, they prefer access through the maxillary sinus to gently push the eyeball back to its original position. Additionally, to preserve the appearance and position of the eyeball, the use of catheters or balloons inside the paranasal sinuses to repair blowout fractures is well-stated. Applying a maxillary sinus balloon is generally beneficial in cases involving more extensive orbital floor fractures that may lead to significant enophthalmos [9–11]. However, in this case, transconjunctival approach facilitated the removal of orbital fracture fragments, repositioning the eyeball, and reconstructing the orbital structure in a single session, ultimately yielding a satisfying clinical outcome.

As for the reconstructing the orbital wall, the choice of autogenous bone grafts or biocompatible alloys remains crucial. After severe orbital trauma, using implants to restore the normal anatomical structure is a common surgical practice. Reconstruction materials, such as autogenous bone transplantation, metals (titanium, vitallium), porous polyethylene, hydroxyapatite, and ceramics, have all shown favourable clinical outcomes [9].

In paediatric patients with OBF, persistent diplopia stands out as the most prevalent postoperative complication [12, 13]. Lin et al. [14] reported that even at 1-month post-surgery, 86% of patients still grappled with diplopia. In addition, the recovery rate at the 1-year mark was merely 78.4%, leaving 21.6% of the patients still experiencing diplopia. Liu et al. [15] noted that patients operated on within 3 days tended to exhibit near-complete diplopia recovery, while those undergoing surgery after 10 days showed a significant disparity in diplopia recovery compared to the early (< 3 days) group. Research indicates that the duration of extraocular muscle entrapment correlates with muscle fibre necrosis, leading to a decline in extraocular muscle function [15].

Furthermore, surgeries performed at a later stage are associated with pronounced fibrosis within the orbit, making it more susceptible to scar adhesions. This, in turn, leads to limitations in postoperative eye movement and persistent diplopia. It is essential to acknowledge that the patient in this study experienced traumatic optic neuropathy (TON). A study suggests that TON occurs in 0.5–7% of head injury cases [16]. Apart from increased intracranial pressure, periorbital tissue oedema compressing the optic nerve, and damage to orbital vessels causing optic nerve ischaemia, the traction on the optic nerve induced by the displacement of the eyeball is also a significant factor. Therefore, an early surgical intervention to restore eyeball position, alleviate entrapment of extraocular muscles or the eyeball to restore blood supply, and mitigate periorbital tissue oedema to prevent optic nerve compression or atrophy is paramount for patients with these conditions.

In this case, we prioritised the patient’s overall well-being and promptly scheduled surgery for eyeball repositioning and orbital reconstruction. Using transconjunctival access, we reached the fracture plane, removed fragmented bone pieces, expanded the fracture hole, and carefully pulled the eyeball back to its original position after reducing resistance. This approach was crucial to minimize the substantial risk of inducing secondary damage during the traction procedure. At the 12-month follow-up, the patient achieved a visual acuity of 20/200. Despite limited eye movement, the overall outcome was deemed satisfactory.

## Data Availability

The data and materials used and analysed during the current study are available from the corresponding author upon reasonable request.

## Abbreviations

CT: computed tomography
GCS: Glasgow Coma Scale
ICU: intensive care unit
OBF: orbital blowout fracture
OCT: optical coherence tomography
TON: traumatic optic neuropathy

## References

[1] Firriolo JM, Ontiveros NC, Pike CM, Taghinia AH, Rogers-Vizena CR, Ganor O (2017). Pediatric orbital floor fractures: clinical and radiological predictors of tissue entrapment and the effect of operative timing on ocular outcomes. J Craniofac Surg.

[2] Chau A, Fung K, Yip L, Yap M (2004). Orbital development in Hong Kong Chinese subjects. Ophthalmic Physiol Opt.

[3] Buck LS, Stockton S, Spankovich C, Jordan JR (2020). Pediatric orbital floor fractures and the oculocardiac reflex: experience from a level I trauma center. Am J Otolaryngol.

[4] Smith B, Regan WF (1957). Blow-out fracture of the orbit; mechanism and correction of internal orbital fracture. Am J Ophthalmol.

[5] Fujino T, Makino K (1980). Entrapment mechanism and ocular injury in orbital blowout fracture. Plast Reconstr Surg.

[6] Hink EM, Wei LA, Durairaj VD (2014). Clinical features and treatment of pediatric orbit fractures. Ophthalmic Plast Reconstr Surg.

[7] Amaral MB, Nery AC (2016). Traumatic globe dislocation into the paranasal sinuses: literature review and treatment guidelines. J Craniomaxillofac Surg.

[8] Noman SA, Shindy MI (2017). Immediate surgical management of traumatic dislocation of the eye globe into the maxillary sinus: report of a rare case and literature review. Craniomaxillofac Trauma Reconstr.

[9] Kashimura T, Soejima K, Kikuchi Y, Nakazawa H (2017). Stability of orbital floor fracture fixation after endoscope-assisted balloon placement. J Craniofac Surg.

[10] Jo EJ, Kim JH, Yang HJ (2015). Inferior blow-out fracture reduction using two urinary balloon catheters. Arch Craniofac Surg.

[11] Soejima K, Shimoda K, Kashimura T, Yamaki T, Kono T, Sakurai H (2013). Endoscopic transmaxillary repair of orbital floor fractures: a minimally invasive treatment. J Plast Surg Hand Surg.

[12] Kansakar P, Sundar G (2020). Vision loss associated with orbital surgery - a major review. Orbit.

[13] Balaraman K, Patnaik JSS, Ramani V, Bhat K, Thomas D, Bharathi RR (2021). Management of white-eyed blowout fracture in the pediatric population. J Maxillofac Oral Surg.

[14] Liu W, Lin M, Shi WD (2021). Application of absorbable plate in the repairment of orbital trapdoor fracture in children. Guoji Yanke Zazhi (Int Eye Sci).

[15] Su Y, Shen Q, Lin M, Fan X (2015). Diplopia of pediatric orbital blowout fractures: a retrospective study of 83 patients classified by age groups. Med (Baltim).

[16] Bastos RM, Taparello C, Tres R, Sawazaki R (2021). Orbital blowout fracture with globe displacement into the maxillary sinus: a case report and literature review. J Oral Maxillofac Surg.

